# P4-ATPases as Phospholipid Flippases—Structure, Function, and Enigmas

**DOI:** 10.3389/fphys.2016.00275

**Published:** 2016-07-08

**Authors:** Jens P. Andersen, Anna L. Vestergaard, Stine A. Mikkelsen, Louise S. Mogensen, Madhavan Chalat, Robert S. Molday

**Affiliations:** ^1^Department of Biomedicine, Aarhus UniversityAarhus, Denmark; ^2^Department of Biochemistry and Molecular Biology, University of British ColumbiaVancouver, BC, Canada

**Keywords:** P-type ATPases, P4-ATPases, ATP8A2, CDC50, flippases, membrane asymmetry, phospholipid transport

## Abstract

P4-ATPases comprise a family of P-type ATPases that actively transport or flip phospholipids across cell membranes. This generates and maintains membrane lipid asymmetry, a property essential for a wide variety of cellular processes such as vesicle budding and trafficking, cell signaling, blood coagulation, apoptosis, bile and cholesterol homeostasis, and neuronal cell survival. Some P4-ATPases transport phosphatidylserine and phosphatidylethanolamine across the plasma membrane or intracellular membranes whereas other P4-ATPases are specific for phosphatidylcholine. The importance of P4-ATPases is highlighted by the finding that genetic defects in two P4-ATPases ATP8A2 and ATP8B1 are associated with severe human disorders. Recent studies have provided insight into how P4-ATPases translocate phospholipids across membranes. P4-ATPases form a phosphorylated intermediate at the aspartate of the P-type ATPase signature sequence, and dephosphorylation is activated by the lipid substrate being flipped from the exoplasmic to the cytoplasmic leaflet similar to the activation of dephosphorylation of Na^+^/K^+^-ATPase by exoplasmic K^+^. How the phospholipid is translocated can be understood in terms of a peripheral hydrophobic gate pathway between transmembrane helices M1, M3, M4, and M6. This pathway, which partially overlaps with the suggested pathway for migration of Ca^2+^ in the opposite direction in the Ca^2+^-ATPase, is wider than the latter, thereby accommodating the phospholipid head group. The head group is propelled along against its concentration gradient with the hydrocarbon chains projecting out into the lipid phase by movement of an isoleucine located at the position corresponding to an ion binding glutamate in the Ca^2+^- and Na^+^/K^+^-ATPases. Hence, the P4-ATPase mechanism is quite similar to the mechanism of these ion pumps, where the glutamate translocates the ions by moving like a pump rod. The accessory subunit CDC50 may be located in close association with the exoplasmic entrance of the suggested pathway, and possibly promotes the binding of the lipid substrate. This review focuses on properties of mammalian and yeast P4-ATPases for which most mechanistic insight is available. However, the structure, function and enigmas associated with mammalian and yeast P4-ATPases most likely extend to P4-ATPases of plants and other organisms.

## Introduction

Non-random distribution of lipids across the lipid bilayer is a characteristic feature of most eukaryotic cell membranes. This asymmetrical distribution is most evident in the plasma membrane where phosphatidylcholine (PC), sphingomyelin, and glycolipids are enriched on the external or exoplasmic leaflet of the membranes and phosphatidylserine (PS), phosphatidylethanolamine (PE), and phosphatidylinositol (PI) are primarily confined to the cytoplasmic leaflet (Zachowski, 1993; Lenoir et al., 2007). Cholesterol, a major lipid of the plasma membrane, can move freely between the two leaflets, but is likely to be more concentrated on the exoplasmic leaflet due to its affinity for sphingomyelin and glycosphingolipids. Membranes of other subcellular organelles including the trans-Golgi network (TGN), secretory vesicles, and endosomes show a similar asymmetrical distribution of lipids. Membrane lipid asymmetry plays a crucial role in numerous cellular processes that take place at the plasma membrane and intracellular membranes including cell and organelle shape determination and dynamics, vesicle budding and trafficking, membrane protein regulation, membrane stability and impermeability, cell signaling, neurite extension, blood coagulation, apoptosis, fertilization, and bile and cholesterol homeostasis, among others.

The amphipathic nature of phospholipids impedes their transverse movement or flip-flop across the hydrophobic lipid bilayer. The half time for phospholipid flip-flop in protein-free liposomes typically exceeds several hours. However, the movement of lipids across biological membranes is greatly accelerated by membrane proteins generally called flippases (Pomorski and Menon, 2006). Three families of proteins, scramblases, ATP-binding cassette (ABC) transporters, and P4-ATPases, have been implicated in the transverse movement of lipids across membranes. Scramblases are energy-independent, bidirectional transporters that dissipate membrane asymmetry (Williamson, 2015). Ca^2+^-activated scramblases play an important role in exposing PS on the surface of cells to initiate such cellular processes as blood coagulation and apoptosis. Some scramblases such as the G-protein coupled receptor rhodopsin are constitutively active thereby randomizing transmembrane lipid distribution in specialized membranes such as photoreceptor discs (Menon et al., 2011). ABC transporters and P4-ATPases use the energy from ATP hydrolysis to transport specific lipids across membranes against their concentration gradient, thereby establishing and maintaining lipid asymmetry in biological membranes (Coleman et al., 2013; Lopez-Marques et al., 2014). Most ABC transporters translocate lipids from the cytoplasmic to the exoplasmic leaflet of membranes and are often called floppases. Some mammalian ABC transporters, however, can transport phospholipids in the opposite direction (Quazi and Molday, 2013). P4-ATPases are a subfamily of P-type ATPases that transport specific phospholipids from the exoplasmic to the cytoplasmic leaflet of membranes to generate and maintain membrane lipid asymmetry (Tang et al., 1996; Coleman et al., 2009; Zhou and Graham, 2009).

A number of excellent reviews have focused on the molecular properties of lipid flippases and their role in cellular processes (Sebastian et al., 2012; Coleman et al., 2013; Lopez-Marques et al., 2013, 2014; Hankins et al., 2015; Williamson, 2015; Montigny et al., 2016). In this review we discuss recent studies on the lipid substrate specificity and transport mechanism of P4-ATPases and their role in cell physiology and disease with emphasis on mammalian and yeast P4-ATPases. We also present an overview of recent site-directed mutagenesis and molecular modeling studies that provide insight into the possible pathways used by P4-ATPases to translocate phospholipids across membranes. Finally, we discuss similarities in the structure and transport mechanisms of P4-ATPase lipid pumps and P2-ATPase ion pumps.

## P-type-ATPases

P-type ATPases constitute a family of membrane proteins that utilize the energy from ATP hydrolysis to transport ions and lipids across biological membranes (Palmgren and Nissen, 2011). These pumps are found in prokaryotes, archaea, and eukaryotes and are distinguished from other ATP-dependent transporters by the presence of a conserved aspartic acid residue that undergoes transient phosphorylation during the ATP cycle (Pedersen and Carafoli, 1987). On the basis of phylogenetic analysis, P-type ATPases have been organized into five main classes (P1-P5 ATPases) (Palmgren and Axelsen, 1998). The first three classes have been further divided into subclasses based on sequence similarity and ion transport specificity. P1A-ATPases are only found in some bacteria and transport K^+^; P1B-ATPases transport heavy metal ions such as Ag^+^, Zn^2+^, Cd^2+^, and Cu^2+^; P2A ATPases (sarco(endo)plasmic reticulum Ca^2+^-ATPase (SERCA) and secretory pathway Ca^2+^-ATPase (SPCA)) and P2B ATPases (plasma membrane Ca^2+^-ATPase (PMCA)) transport Ca^2+^ and Mn^2+^; P2C-ATPases transport monovalent ions and include Na^+^/K^+^-ATPases and H^+^/K^+^-ATPases; P2D-ATPases are found in fungi and transport Na^+^; P3A-ATPases transport H^+^; and P3B ATPases transport Mg^2+^. P4-ATPases are unique in that they transport or flip phospholipids across membranes. The substrates for P5-ATPases have yet to be determined, but they are predicted to play an important role in the endosomal-lysosomal system and have been implicated in Parkinson's disease (Schultheis et al., 2004; Cohen et al., 2013; De La Hera et al., 2013).

## P4-ATPases

**Figure 1:**
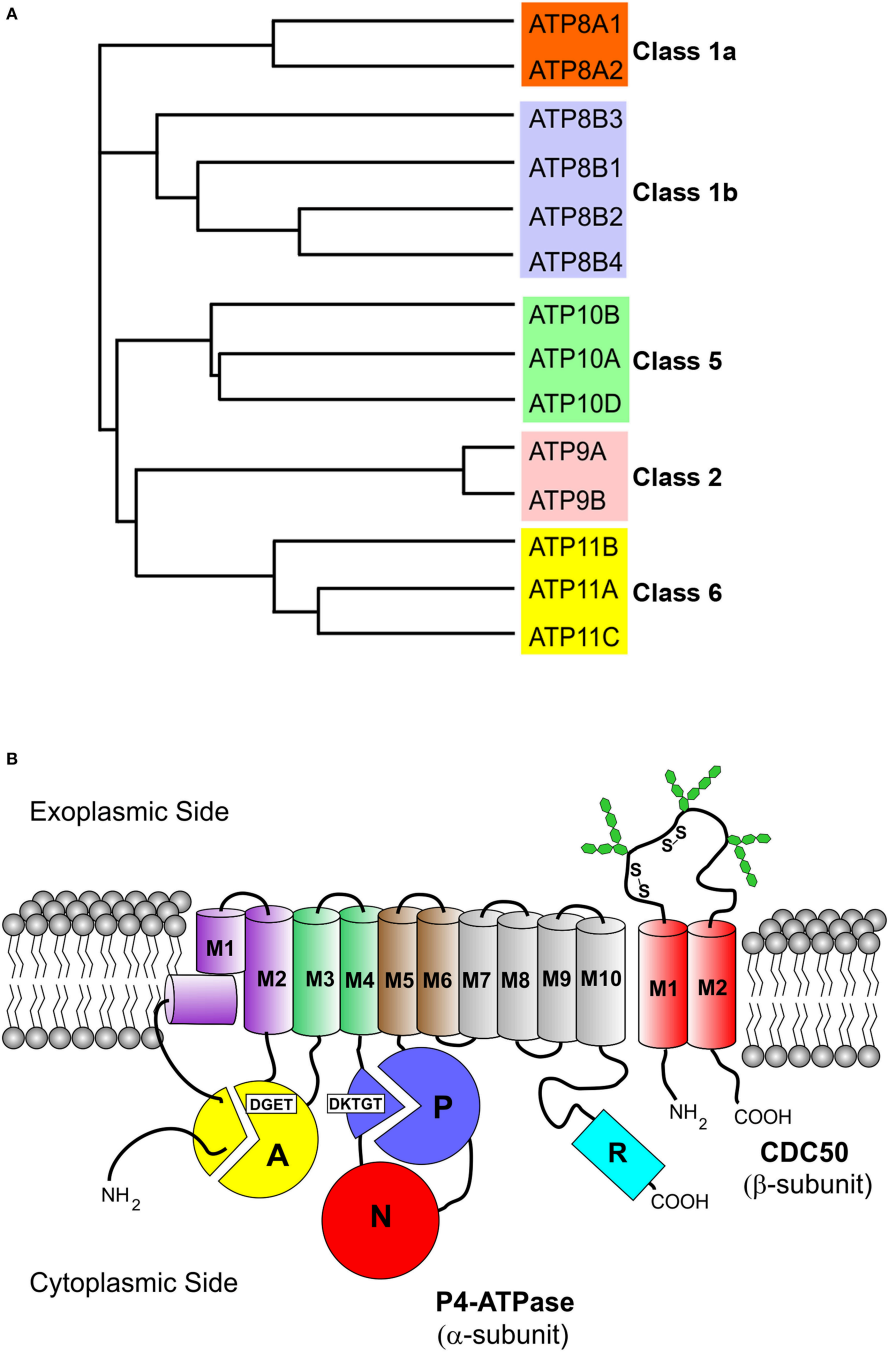
**Phylogenetic analysis and membrane topology of P4-ATPases. (A)** The sequences of the 14 human P4-ATPases were aligned using the Clustal Omega multi-sequence alignment program for generation of the phylogenetic tree. Protein sequences are from the following accession numbers: ATP8A1 (Q9Y2Q0); ATP8A2 (Q9NTI2); ATP8B1 (O43520); ATP8B2 (P98198); ATP8B3 (O60423); ATP8B4 (Q8TF62); ATP9A (O75110); ATP9B (O43861); ATP10A (O60312); ATP10B (O94823); ATP10D (Q9P241); ATP11A (P98196); ATP11B (Q9Y2G3); ATP11C (Q8NB49); **(B)** The cytoplasmic A (“actuator”), N (nucleotide binding), and P (phosphorylation) domains of the α-subunit are shown as colored circles. Conserved motifs involved in phosphorylation (DKTGT) and dephosphorylation (DGET) are indicated for the respective domains. The 10 transmembrane helices of the α-subunit are represented by cylinders (M1 kinked), M1–M2 purple, M3–M4 green, M5–M6 brown, and M7–M10 gray. The two transmembrane helices of the β-subunit (CDC50 protein) are shown as red cylinders. Glycosylation and disulfide bridges of the exoplasmic domain of the β-subunit are also indicated. The C-terminal cytoplasmic extension of the α-subunit furthermore contains a regulatory domain shown as cyan cylinder (R domain).

P4-ATPases are only found in eukaryotes. The human genome encodes 14 P4-ATPases that are organized into five classes with each class having multiple members (Table 1, Figure 1A): Class 1a (ATP8A1, ATP8A2); Class 1b (ATP8B1, ATP8B2, ATP8B3, ATP8B4); Class 2 (ATP9A, ATP9B); Class 5 (ATP10A, ATP10B, ATP10D); and Class 6 (ATP11A, ATP11B, ATP11C) (Paulusma and Elferink, 2010; Van Der Mark et al., 2013). In contrast, yeast (*Saccharomyces cerevisiae*) contains five P4-ATPases (Drs2p, Neo1p, Dnf1p, Dnf2p, Dnf3P).

**Table 1:** **Mammalian P_4_-ATPases**.

**Class**	**P_4_-ATPase Complex (α/β)**	**Substrate**	**Expression**	**Disease/Disorder**	**References**
Class 1a	ATP8A1/CDC50A	PS >PE	Ubiquitous, high in skeletal muscle, thyroid, spinal cord	Defective hippocampus-dependent learning (mice)	Levano et al., 2012; Kato et al., 2013; Lee et al., 2015
	ATP8A2/CDC50A	PS > PE	High in brain, retina, testis, spinal cord	Cerebellar Ataxia, Mental Retardation and Disequilibrium Syndrome (CAMRQ) (humans) Neurological degeneration, spinal, axonal degeneration; retinal degeneration; hearing loss (mice)	Coleman et al., 2009, 2014; Onat et al., 2012; Zhu et al., 2012; Vestergaard et al., 2014
Class 1b	ATP8B1/CDC50A ATP8B1/CDC50B	PC (PS?)	Ubiquitous, high in small intestine, pancreas	Progressive Familial Intrahepatic Cholestasis (PFIC); Benign Recurrent Intrahepatic Cholestasis (BRIC) (human) Intrahepatic cholestasis, hearing loss, (mice)	Bull et al., 1998; Eppens et al., 2001; Paulusma et al., 2006; Stapelbroek et al., 2009; Folmer et al., 2009b; Takatsu et al., 2014
	ATP8B2/CDC50A ATP8B2/CDC50B	PC	Ubiquitous	Unknown	Takatsu et al., 2014
	ATP8B3/??	PS?	Testis	Abnormal sperm-egg interactions	Wang et al., 2004; Gong et al., 2009
	ATP8B4/CDC50A ATP8B4/CDC50B?	Unknown	Moderate levels throughout brain	Alzheimer disease?	Li et al., 2008; Van Der Velden et al., 2010b
	ATP8B5 (FetA)	Unknown	Testis	Defects in sperm capacitation	Xu et al., 2009
Class 2	ATP9A	Unknown	Ubiquitous, high in brain, pancreas	Unknown	Takatsu et al., 2011; Ansari et al., 2015
	ATP9B	Unknown	Ubiquitous, high in testis	Unknown	Takatsu et al., 2011
Class 5	ATP10A/CDC50A	PC	High in brain, pancreas, kidney, lung	Obesity, type 2 diabetes, insulin resistance	Dhar et al., 2004, 2006; Naito et al., 2015
	ATP10B/CDC50A	Unknown	Low expression, brain	Unknown	
	ATP10D/CDC50A	Unknown	High in placenta, low kidney, undetectable in other major organs	Obesity; hyperinsulinemia	Flamant et al., 2003; Takatsu et al., 2011
Class 6	ATP11A/CDC50A	PS > PE	Ubiquitous, moderate levels in liver, skeletal muscle, ovary	Marker for metastasis in colorectal cancer (human)	Miyoshi et al., 2010; Takatsu et al., 2014
	ATP11B/CDC50A	PS > PE	Ubiquitous, high levels in kidney, testis, ovary	Unknown	
	ATP11C/CDC50A	PS > PE	Ubiquitous, high liver, pancreas, heart	Impaired B lymphocyte differentiation, cholestasis, hepatocarcinoma; anemia, altered erythrocyte shape (mice)	Siggs et al., 2011b;Takatsu et al., 2014; Yabas et al., 2014

**Figure 2:**
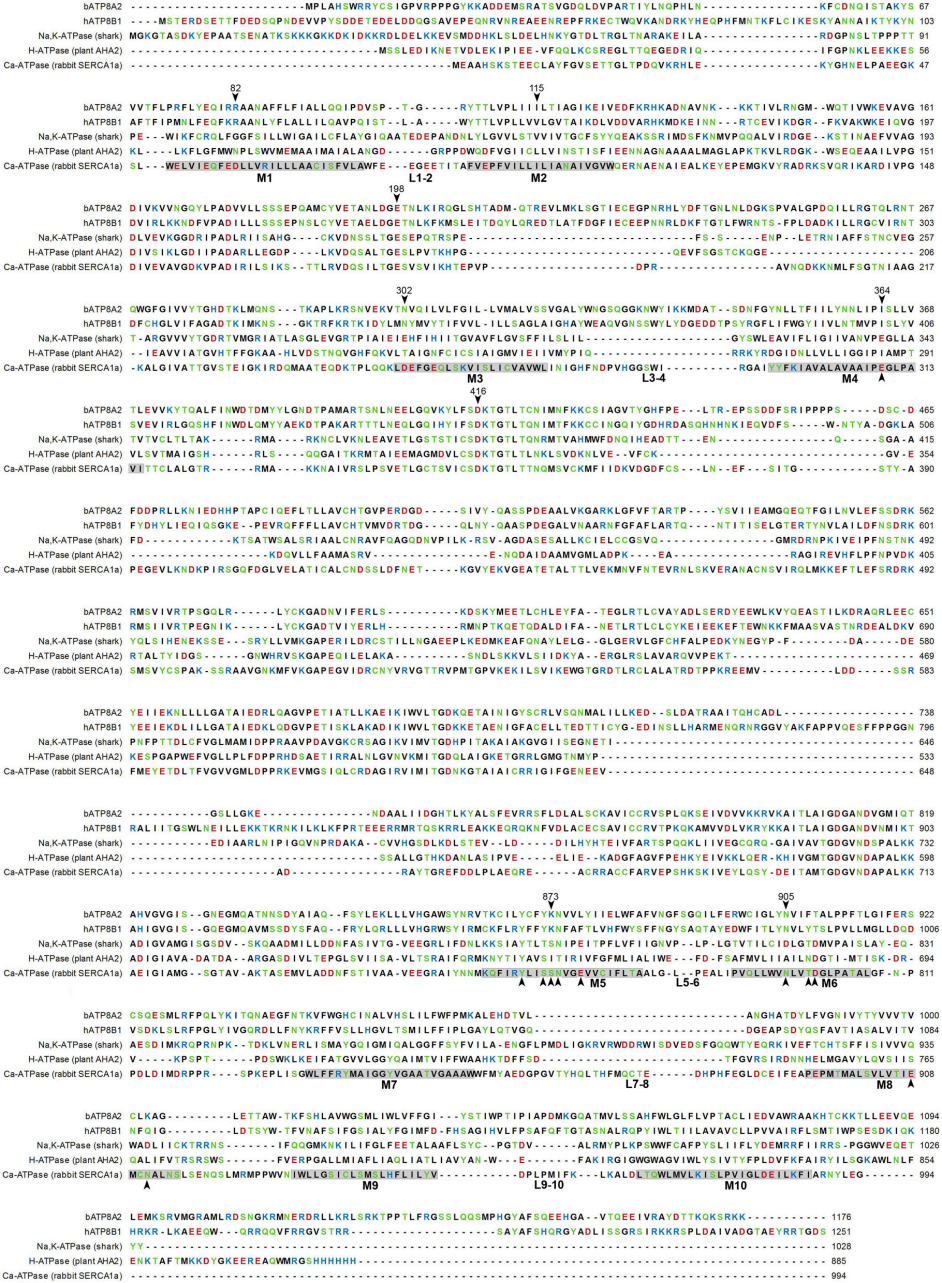
**Amino acid sequence alignment of P4-ATPases and P-type ion pumps**. Sequences of two P4-ATPases, bovine ATP8A2 (UniProt: C7EXK4) and human ATP8B1 (UniProt: O43520), are shown aligned with sequences of the P2- and P3-ATPases shark Na^+^/K^+^-ATPase (UniProt: Q4H132, PDB: 2ZXE), plant AHA2 H^+^-ATPase (UniProt: P19456, PDB: 3B8C), and sarco(endo)plasmic reticulum Ca^2+^-ATPase (SERCA) isoform 1a (UniProt: P04191, PDB: 3B9B). Letter colors denote positively (blue) or negatively (red) charged, polar (green), or hydrophobic (black) residues. Membrane parts of transmembrane helices of SERCA identified in the crystal structure are indicated by gray shading with helix numbering and indication of exoplasmic loops under the sequence. Arrowheads with residue number above the bATP8A2 sequence indicate some of the residues that are mentioned in the text or shown in the figures. Arrowheads below the SERCA sequence indicate residues of SERCA or the Na^+^/K^+^-ATPase involved in binding the ions transported by these ATPases. This alignment was based on a previously described multiple sequence alignment (Vestergaard et al., 2014).

All P4-ATPases consist of a large polypeptide with a molecular mass of approximately 120 kDa which is composed of four main domains (Figure 1B). Based on amino acid sequence alignment (Figure 2), P4-ATPases possess three cytoplasmic domains involved in the ATPase catalytic cycle similar to Ca^2+^-ATPase and Na^+^,K^+^-ATPase: the nucleotide or N-domain binds ATP; the phosphorylation or P-domain contains the aspartic acid (D) residue within the conserved DKTGT motif that undergoes transient phosphorylation; and the actuator or A-domain has the DGET (TGES in Ca^2+^-ATPase and Na^+^/K^+^-ATPase) motif that facilitates the dephosphorylation of the phosphorylated intermediate (Lenoir et al., 2009; Coleman et al., 2012). The membrane or M-domain serves as the pathway for translocation of lipid substrates across cell membranes and is predicted to contain 10 transmembrane segments (M1-M10, Figure 2) analogous to the Ca^2+^-ATPase and the Na^+^/K^+^-ATPase (Maclennan et al., 1985; Toyoshima et al., 2000; Morth et al., 2007). Relatively short segments join the transmembrane segments on the exoplasmic side of the membrane, whereas large domains and segments connect some of the transmembrane segments on the cytoplasmic side (Figures 1, 2). Recent studies suggest that transmembrane segments M1–M6 form the principal unit for transport of phospholipids across membranes with M7–M10 playing a supporting role (Baldridge and Graham, 2012; Vestergaard et al., 2014). P4-ATPases also contain regulatory domains that modulate the transport activity and targeting domains that participate in the trafficking of the P4-ATPases to their preferred subcellular membranes. These domains are present, at least in part, along the cytoplasmic C-terminal and N-terminal segments of P4-ATPases, but to date are poorly characterized for mammalian P4-ATPases (Takatsu et al., 2011; Zhou et al., 2013).

## CDC50–accessory subunit

P4-ATPases with the exception of ATP9A and ATP9B assemble with an accessory or β-subunit to form a heteromeric complex, most likely a heterodimer. The β-subunit belongs to the CDC50/LEM3 family of evolutionary conserved proteins present in all eukaryotes (Katoh and Katoh, 2004; Saito et al., 2004). There are three CDC50 proteins (CDC50A, CDC50B, and CDC50C, also known as TMEM30A, TMEM30B, and TMEM30C) in humans and three in yeast (Cdc50p, Lem3p, Crf1p). Since there are more P4-ATPases (α-subunits) than CDC50 proteins (β-subunits), many P4-ATPases associate with the same CDC50 protein to form the heteromeric complex (Bryde et al., 2010).

The interaction of mammalian P4-ATPases with specific CDC50 proteins has been studied primarily by heterologous protein co-expression and co-immunoprecipitation (Paulusma et al., 2008; Bryde et al., 2010; Van Der Velden et al., 2010b; Coleman and Molday, 2011; Takatsu et al., 2011, 2014; Naito et al., 2015). These studies indicate that most mammalian P4-ATPases use CDC50A as their β-subunit. These include ATP8A1, ATP8A2, ATP11A, ATP11B, ATP11C, ATP10A, ATP10B, and ATP10D. Several P4-ATPases including ATP8B1, ATP8B2, and ATP8B4 can associate with either CDC50A or CDC50B. The physiological consequence of this dual specificity, however, remains to be determined. CDC50C is predominantly expressed in testes (Osada et al., 2007), but to date it has not been shown to associate with any P4-ATPase. Although, heterologous overexpression provides useful information on the interaction of P4-ATPases with specific CDC50 proteins, the presence of such complexes needs to be confirmed in physiologically relevant cells and tissues. In one study ATP8A2 was found to interact with CDC50A in photoreceptors in support of heterologous cell expression and immunoprecipitation studies (Coleman et al., 2009; Coleman and Molday, 2011).

CDC50 proteins are relatively small membrane glycoproteins containing approximately 350 amino acids. They consist of two transmembrane segments joined by a large exoplasmic domain with two or more N-linked oligosaccharide chains that contribute to the stability of the protein (Coleman and Molday, 2011; Garcia-Sanchez et al., 2014; Figure 1B). CDC50 proteins typically migrate on SDS gels as a broad band with an approximate molecular mass of 50 kDa due to the highly heterogeneous nature of the oligosaccharide chains. The exoplasmic domain also contains four conserved cysteine residues that stabilize the structure through the formation of two disulfide bonds (Puts et al., 2012). Relatively, short N-terminal and C-terminal segments are exposed on the cytoplasmic side of the membrane.

Heterologous expression studies point to the crucial role of CDC50 in protein folding and formation of a functionally active P4-ATPase complex (Paulusma et al., 2008; Bryde et al., 2010; Van Der Velden et al., 2010b; Coleman and Molday, 2011). In the absence of CDC50 expression, P4-ATPases fail to exit the ER and are incapable of undergoing phosphorylation or phospholipid-dependent ATP hydrolysis (Lopez-Marques et al., 2010; Coleman and Molday, 2011; Puts et al., 2012). Likewise, in the absence of P4-ATPase expression, CDC50A is retained in the ER of cells. However, co-expression of P4-ATPases and CDC50 results in a P4-ATPase-CDC50 complex that can exit the ER and function as an active phospholipid flippase (Coleman and Molday, 2011). CDC50 proteins do not play a direct role in the targeting of P4-ATPases to their preferred subcellular location once they exit the ER in multicellular organisms (Lopez-Marques et al., 2010).

Several studies suggest that CDC50 proteins participate in ATP-dependent phospholipid transport. The affinity of yeast Drs2p for Cdc50p fluctuates during the catalytic reaction cycle with the highest affinity observed for Drs2p in the E2P state with a bound phospholipid substrate (Lenoir et al., 2009). Phosphorylation of Drs2p, ATP8B1, and ATP8B2 are dependent on interaction with their CDC50 subunit (Lenoir et al., 2009; Bryde et al., 2010). Studies using mammalian CDC50A/CDC50B chimeric proteins further indicate that both the transmembrane and exoplasmic domains of CDC50A are necessary for the binding CDC50A to ATP8A2 and the formation of an active complex (Coleman and Molday, 2011). The N-terminal cytoplasmic domain of CDC50A also modulates the kinetics of ATP8A2 ATPase activity.

**Figure 3:**
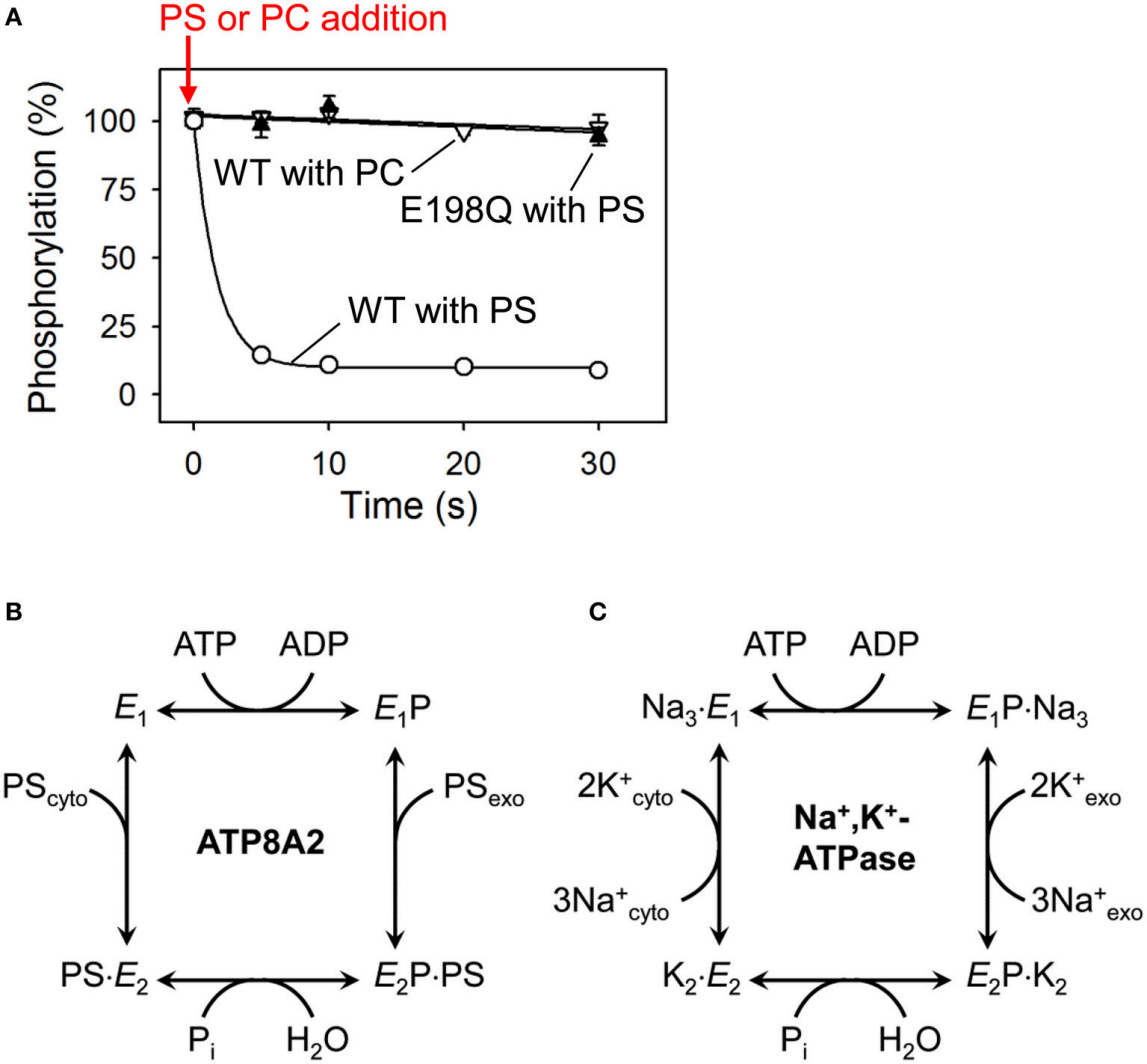
**Dephosphorylation of P4-ATPases and Na^+^/K^+^-ATPase is activated by binding of the transported substrate. (A)** Experimental data (Coleman et al., 2012) showing the accelerating effect of addition of phosphatidylserine (PS) on dephosphorylation of ATP8A2 (open circles). No dephosphorylation occurs when phosphatidylcholine (PC) is added (open triangles). The PS-induced dephosphorylation is blocked by the E198Q mutation replacing the glutamate in the DGET motif of the A domain with glutamine (filled triangles). **(B)** Reaction cycle of P4-ATPases proposed on the basis of the finding illustrated in A together with additional analysis of the conformations of the phosphoenzyme intermediate of ATP8A2 (Coleman et al., 2012). **(C)** Classic Post-Albers model for the reaction cycle of Na^+^,K^+^-ATPase (Post et al., 1972) illustrating that the activation of dephosphorylation of the P4-ATPase by PS (coming from the exoplasmic lipid bilayer leaflet) shown in **(A,B)** is analogous to the activation of dephosphorylation of Na^+^,K^+^-ATPase by K^+^ (coming from the exoplasmic medium). Na^+^ binding from the cytoplasm is required for phosphorylation of Na^+^,K^+^-ATPase, whereas no known ion or other substrate needs to bind to the P4-ATPase to allow phosphorylation (see text for more explanation).

Certain mutations in the transmembrane segments of ATP8A2 decrease glycosylation of CDC50 without significantly affecting the functional properties of the complex and seem to define a groove between M1, M3, and M4 of ATP8A2 as a possible location of CDC50 interactions (Vestergaard et al., 2015; see later text in relation to **Figure 6**). Altogether, the available information indicates that CDC50 accessory proteins contact P4-ATPases at multiple sites to promote correct protein folding, exit of the P4-ATPase complex from the ER, and formation of a functionally active flippase complex. Hence, CDC50 proteins appear to play a role reminiscent of the β-subunits of Na^+^/K^+^-ATPases (Puts and Holthuis, 2009). Because E2P is the state, in which the P4-ATPase is supposed to bind its phospholipid substrate from the exoplasmic membrane leaflet (see reaction cycle in Figure 3B), the preferential interaction of the CDC50 protein with E2P (Lenoir et al., 2009) is consistent with participation of CDC50 in substrate loading analogous to the role of the β-subunit of Na^+^/K^+^-ATPase in K^+^ binding (Shinoda et al., 2009). Nevertheless, CDC50 and the β-subunit of Na^+^/K^+^-ATPase differ with respect to the number of transmembrane segments (two for CDC50 and only one for the Na^+^/K^+^-ATPase β-subunit), the phylogenetic origin, and apparently also the α-subunit contact sites (M7 and M10 for the Na^+^/K^+^-ATPase; a groove between M1, M3, and M4 for ATP8A2).

Class 2 P4-ATPases (ATP9A and ATP9B in mammals and Neo1p in yeast) do not appear to use CDC50 proteins as β-subunits. Both ATP9A and ATP9B exit the ER and localize to their respective cell membranes in the absence of CDC50 proteins (Takatsu et al., 2011). Neo1p is the only yeast P4-ATPase that is lethal when its gene is deleted pointing to its essential role in yeast survival (Prezant et al., 1996; Hua et al., 2002). Neo1p forms a complex with Ysl2p, a guanine nucleotide exchange factor for Arl1p that functions in vesicle trafficking within the Golgi/endosomal system (Wicky et al., 2004; Barbosa et al., 2010). The function of ATP9A and ATP9B is not known and it remains to be determined if these P4-ATPases require an accessory subunit or associated protein for activity and whether they transport phospholipids across membranes.

## Distribution of P4-ATPases in cells

The subcellular localization of mammalian P4-ATPases has been mostly studied by immunofluorescence microscopy and transport of fluorescently labeled phospholipids into cells overexpressing the protein complex. Although, there is some variation possibly due to the cell type used or extent of overexpression, in general several P4-ATPases including ATP11C, ATP11A, ATP10A, ATP10D, ATP8B1, ATP8B2, and ATP8B4 in association with their β-subunit preferentially localize to the plasma membrane of cells where they transport specific phospholipids from the extracellular to the cytoplasmic leaflet (Van Der Velden et al., 2010b; Takatsu et al., 2011, 2014; Naito et al., 2015). Of these P4-ATPases, ATP11C has been widely studied and shown to play a critical role in the internalization of PS thereby preventing its exposure on cell surfaces (Yabas et al., 2011, 2014; Segawa et al., 2014). The P4-ATPases ATP8A1, ATP8A2, ATP10B, and ATP11B are largely confined to Golgi/recycling endosomal system when overexpressed in culture cells (Coleman and Molday, 2011; Takatsu et al., 2011; Lee et al., 2015). However, in some studies, several of these P4-ATPases have been reported to be also present on the plasma membrane of cells (Segawa et al., 2016). ATP8A2 has been shown to transport fluorescently labeled PS into cells and prevent exposure of PS on cell surfaces. ATP8A1 localizes to the plasma membrane of erythrocytes (Soupene and Kuypers, 2006). Taken together, these studies indicate that P4-ATPases can cycle between the Golgi/endosomal recycling system and the plasma membrane, but preferentially localize to either the plasma membrane or Golgi-recycling endosome network depending on the specific P4-ATPase.

The subcellular localization of the Class 2 P4-ATPases, ATP9A and ATP9B, has also been studied by overexpression in Hela cells (Takatsu et al., 2011). ATP9A localizes to early/recycling endosomes and the trans-Golgi network (TGN), whereas ATP9B localizes exclusively to the TGN. A chimeric ATP9 protein in which the N-terminal cytoplasmic region of ATP9A was replaced with the corresponding region of ATP9B localized exclusively to the TGN indicating that a segment within the N-terminal cytoplasmic region of ATP9 proteins is responsible for subcellular localization. ATP9A is highly expressed in neural and pancreatic tissue whereas ATP9B is more ubiquitously expressed. Recently, ATP9A has been reported to be present in human and rat pancreatic islets and based on membrane fractionation appears concentrated in the plasma membrane (Ansari et al., 2015). ATP9A, like other P4-ATPases may cycle between the TGN and plasma membrane of cells.

It should be noted, however, that protein overexpression can result in abnormal localization in cells. Accordingly, it will be important to examine the distribution of endogenous P4-ATPases in physiologically relevant tissues and cells. A set of highly specific antibodies to the various P4-ATPases would be particularly valuable to probe their distribution in various cells and tissues.

## Phospholipid substrate specificity

The substrate specificities for only a few mammalian P4-ATPases have been determined at a biochemical level with most being specific for the aminophospholipids, PS and PE (Table 1). Historically, aminophospholipid translocase (APLT) activity was first found in red blood cells and bovine chromaffin granules (Moriyama and Nelson, 1988; Morrot et al., 1990; Zimmerman and Daleke, 1993; Auland et al., 1994; Tang et al., 1996). The membrane protein responsible for this activity was identified as ATPaseII (now known as ATP8A1) (Mouro et al., 1999; Paterson et al., 2006). Expression in insect cells and yeast confirmed the activation of the ATPase activity of ATP8A1 by PS and more moderate activation by PE (Ding et al., 2000; Paterson et al., 2006; Soupene et al., 2008). More recently, ATP8A1 expressed and purified from HEK293 cells and reconstituted into liposomes has been confirmed to flip PS and to a lesser extent PE across membranes (Lee et al., 2015). While ATP8A1 is widely expressed in different mammalian tissues, the second member of the ATP8A subgroup, ATP8A2, is most highly expressed in retina, brain, spinal cord, and testes (Coleman et al., 2009; Cacciagli et al., 2010; Zhu et al., 2012). ATP8A2 has been purified from retinal photoreceptor cells and transfected HEK293 cells for analysis of its ATPase and phospholipid flippase activity (Coleman et al., 2009, 2012; Coleman and Molday, 2011). Like ATP8A1, ATP8A2 preferentially transports PS across membranes, but can also uses PE as a substrate. Functional complementation experiments further show that ATP8A2 can rescue defects in ATP8A1-depleted cells indicating that ATP8A1 and ATP8A2 share overlapping functional activities (Lee et al., 2015).

While the PS substrate specificity of the ATP8A subclass of P4-ATPases has been confirmed by multiple investigations, the results with respect to the ATP8B proteins have been less definitive. ATP8B1 has been implicated in hepatic disorders such as progressive familial intrahepatic cholestasis (PFIC) and benign recurrent intrahepatic cholestasis (BRIC) (Bull et al., 1998; Eppens et al., 2001). As a result, it has been one of the most studied of the mammalian P4-ATPases. Initial evidence based on increased biliary excretion of PS in ATP8B1 deficient mice, plasma membrane uptake of fluorescent PS, and higher exposure of PS on the outer plasma membrane leaflet in the absence of an active flippase complex implicated ATP8B1 in the flipping of PS across the plasma membrane (Paulusma et al., 2006, 2008). But the results presented in a more recent study point toward PC as being the primary transport substrate of ATP8B1 (Takatsu et al., 2014). The same study also found that ATP8B1 mediated uptake of a NBD-labeled PC analog could be negated by concomitant expression of ABCB4, an ABC transporter that mediates ATP-dependent transport of phospholipids to the extracellular leaflet of plasma membranes. The substrate specificity of the other ATP8B members has not been studied at a biochemical level. Cell based studies, however, suggest that ATP8B2 like ATP8B1 transports PC (Takatsu et al., 2014). ATP8B3 has been implicated in the transport of PS, but the substrate specificity for this flippase requires further study (Wang et al., 2004; Gong et al., 2009). ATP8B5 is present in mice but not humans, and has been implicated in spermatogenesis, but the phospholipid substrate remains to be determined (Xu et al., 2009).

The structural basis for the reported difference in substrate specificity between Class 1a and Class1b P4-ATPases has not been elucidated. Although, the amino acid sequences of the transmembrane segments of ATP8A2 and ATP8B1 only exhibit 33% sequence identity, most of the positively charged residues in ATP8A2 that may interact preferentially with the net negatively charged head group of PS are conserved in ATP8B1 (Figure 2, Naito et al., 2015). Subtle structural differences of the α subunits, matching the translocation pathway with the size and hydrogen bonding pattern, rather than the net charge of the head group, could be decisive for the substrate specificity, which might also depend on differences in the interaction of the α-subunit with the CDC50 protein. It is of note that most of the amino acid residues with a suggested role in determining the respective PS and PC specificities of the yeast flippases Drs2p and Dnf1p (Baldridge and Graham, 2012, 2013) are not conserved in the mammalian P4-ATPases with corresponding substrate specificity (Naito et al., 2015).

The substrate specificity of ATP11A and ATP11C has also been investigated (Takatsu et al., 2014). These P4-ATPases, like ATP8A1 and ATP8A2, flip PS as their primary substrate confirming earlier results showing that ATP11C is involved in PS translocation into B-lymphocytes and other immune cells (Yabas et al., 2011, 2016; Segawa et al., 2014). Substrate specificity of ATP10A has recently been reported (Naito et al., 2015). Exogenously expressed ATP10A localizes to the plasma membrane in HeLa cells and produces a significant increase in the uptake of fluorescent NBD-PC, but not other phospholipids. This internalization of PC was not observed in cells expressing an ATPase deficient form of ATP10A.

It needs to be pointed out that aside from ATP8A1 and ATP8A2 reports about the transport substrates of most other mammalian P4-ATPases are based on the ability of cells expressing these proteins to ingest fluorescent phospholipid analogs. This experimental setup is open to the introduction of some confusion regarding the exact nature of the substrate specificity of flippases as seen with the yeast plasma membrane flippases. Greater clarity in this regard is expected to evolve in the future from biochemical characterization of purified proteins.

P4-ATPases expressed in the yeast, *Saccharomyces cerevisiae*, historically have been the most well characterized members of the P4-ATPase family. Drs2p, one of the first phospholipid flippases to be identified, localizes to the TGN and endosomes, where it transports PS and PE from the lumenal to the cytoplasmic leaflet of membranes. Characterization of Drs2p flippase activity was initially based on the results of experiments which looked at the transport of fluorescent (NBD)-labeled PS and PE analogs across TGN membranes purified from yeast strains expressing either the wild type or a temperature sensitive Drs2p mutant (Natarajan et al., 2004). These results were confirmed in direct flippase assays using purified and reconstituted Drs2p (Zhou and Graham, 2009). The TGN membrane shows expression of another P4-ATPase, Dnf3p, which is also involved in transmembrane transport of PS and PE as determined from the loss of asymmetric distribution of fluorescent analogs in the secretory vesicles of dnf3Δ cells (Alder-Baerens et al., 2006). Loss of both Drs2p and Dnf3p dissipates the asymmetric distribution of aminophospholipids in the membranes of TGN and secretory vesicles. Recent mutational analysis has revealed the ability of Drs2p to distinguish between the mono- and di-acylated phospholipid substrate molecules (Baldridge et al., 2013). On the other hand, the two yeast flippases that localize to the plasma membrane, Dnf1p and Dnf2p, have been implicated in the flipping of PE and PC (Pomorski et al., 2003; Stevens et al., 2008). Although, the initial study had suggested that these two proteins might have a role in maintaining the asymmetric distribution of PS in the plasma membrane, it was subsequently shown that they are not essential for this function. In fact, the study by Stevens et al. (2008) found that net internalization of PS increases in dnf1Δdnf2Δ yeast strain, probably due to the enhanced activity of other flippases.

## Regulation of P4-ATPase activity

The mechanism of P4-ATPase regulation has been examined most thoroughly in yeast. The activity of Drs2p is regulated by multiple mechanisms depending on the particular endomembrane system. The C-terminal domain of Drs2p contains a segment of basic amino acids (*RMKKQR*) that has sequence homology to a split PH domain (Natarajan et al., 2009). Drs2p interacts with the phosphatidylinositol-4-phosphate (PI4P) through this domain and this interaction is required for PS flippase activity (Natarajan et al., 2009). The PI4P binding domain in the C-terminal domain of Drs2p overlaps with the binding site of another regulatory element of flippase activity, the Arf-GEF protein Gea2p (Chantalat et al., 2004; Natarajan et al., 2009). As in the case of PI4P, the activity of Drs2p is abolished in the absence of interactions with Gea2p. The requirement for binding of these two regulatory molecules for activation of Drs2p is removed upon cleavage of the C-terminal domain of the protein (Zhou et al., 2013). It has been proposed that the C-terminal domain of Drs2p acts as an auto-inhibitory domain by interfering with the catalytic domains of the protein. Binding of either PI4P or Gea2p disengages the auto-inhibitory domain from the catalytic domain resulting in Drs2p flippase activity. Along with binding to Gea2p at the C-terminal domain, Drs2p also binds to Arl1p at the N-terminal domain. Formation of this trimeric complex is required for flippase activity and for recruitment of cargo during vesicle formation at the TGN (Tsai et al., 2013). While Drs2p interacts with Gea2p at the TGN, it interacts with the F-box protein Rcy1p at an overlapping domain in early endosomes. This interaction is required for the recycling of cargo between endosomes and the TGN (Hanamatsu et al., 2014). The early endosome to TGN retrieval pathway also includes the interaction between Drs2p and Arf-GAP protein Gcs1p (Sakane et al., 2006). The two different interactions of Drs2p with Rcy1 and Gcs1 at the early endosomes may be required for the recruitment of different types of adaptor molecules during the formation of recycling vesicles destined to different membranes.

Apart from the binding of regulatory molecules, the activity of P4-ATPases is also regulated by phosphorylation and sphingolipid concentration. The activity of yeast plasma membrane localizing P4-ATPases, Dnf1p and Dnf2p, requires phosphorylation by kinase proteins, Fpk1 and Fpk2 (Nakano et al., 2008). Deletion mutants for both Fpk1 and Fpk2 (*fpk1*Δ*fpk2*Δ) eliminated the plasma membrane uptake of NBD-labeled phospholipids though the flippases localized normally to the membrane. The activity of Fpk1 and Fpk2 is further regulated by the kinase, Ypk1p. Hence, Ypk1-catalyzed phosphorylation of Fpk1/2 eliminates the activity of these proteins, and the activity of Ypk1 in turn is inhibited due to the phosphorylation by Fpk1/2 proteins. The activity of Fpk1/2 proteins is stimulated by presence of sphingolipids in the membrane. Thus, membrane sphingolipids positively modulate the flippase activity of Dnf1p and Dnf2p (Roelants et al., 2010). Apart from Ypk1, the flippase activity of Dnf1p and Dnf2p is also negatively controlled by Gin4p which phosphorylates Fpk1 to suppress its activity (Roelants et al., 2015).

Little is known about the regulation of mammalian P4-ATPase flippase activity. From limited studies, we know that the flippase activities of ATP11A and ATP11C are down regulated by exposure to calcium with an IC50 in the 100–200 μM range (Segawa et al., 2016). The activity of these two P4-ATPases has also been shown to be suppressed by caspase mediated cleavage. It is hypothesized that the aminophosopholipid translocation activity at the plasma membrane is inactivated by this caspase route during apoptotic exposure of PS (Segawa et al., 2014, 2016). The effect of other regulatory mechanisms including kinase-catalyzed phosphorylation, calcium sensor proteins, and lipids remains to be investigated.

## Role of P4-ATPases in cell physiology

P4-ATPases play a critical role in many cellular processes associated with the plasma membrane and intracellular membranes. A key function of P4-ATPases is to establish and maintain phospholipid asymmetry across membranes. This is most evident for P4-ATPases that utilize PS and PE as substrates. These P4-ATPases including ATP11 and ATP8 subclasses ensure that the extracellular leaflet of the plasma membrane is devoid of PS since exposure of this phospholipid on cell surfaces is an “eat me” signal for ingestion by phagocytic cells. PS and PE specific P4-ATPases also ensure that the cytoplasmic leaflet of membranes is highly enriched in these aminophospholipids. This enables cytoskeletal components, adaptor proteins and enzymes to bind to these membranes where they can function in any of a variety of processes such as cell signaling pathways, enzyme regulation, metabolism, vesicle trafficking and cell motility (Newton and Keranen, 1994; Huang et al., 2011; Lee et al., 2015). The role of PC specific P4-ATPases is less clear, but the active transport of PC to the cytoplasmic leaflet may be important for maintaining the integrity of specialized membranes and/or recruitment of specific PC binding proteins to the cytoplasmic surface under defined conditions.

Although, the main function of P4-ATPases is to actively transport phospholipids across membranes, it is possible that some cellular functions of P4-ATPases are ATP-independent (Van Der Velden et al., 2010a). Other P-type ATPases including Na^+^/K^+^ ATPases are known to bind intracellular and extracellular proteins and hence serve as a scaffold for protein-protein interactions distinct from their ATP-dependent ion transport function (Xie and Cai, 2003; Liang et al., 2007; Molday et al., 2007). ATP-independent functions of P4-ATPases have not been explored in detail, but it is plausible that some P4-ATPases may serve as scaffolds for cell signaling or vesicle trafficking through protein-protein interactions. An overview of some key cellular functions of some mammalian P4-ATPases is given below.

## PS-asymmetry in blood cells

The erythrocyte plasma membrane has been most extensively studied and shown to exhibit a high degree of aminophospholipid asymmetry (Daleke and Huestis, 1989). Early studies showed that ATP8A1 is present in mature erythrocytes and as a result this P4-ATPase was thought to be crucial in restricting PS to the inner leaflet of erythrocytes (Soupene and Kuypers, 2006). However, PS was not exposed on the surface of erythrocytes of ATP8A1-deficient mice and the shape of erythrocytes appeared normal suggesting that increased expression of other P4-ATPases including ATP8A2 may compensate for the absence of ATP8A1 (Levano et al., 2012). Exposure of PS on the external surface of hippocampal neurons of ATP8A1 knockout mice, however, was observed, which correlates with impaired hippocampus-dependent learning. These studies suggest that some cells express other P4-ATPases that can compensate for the loss of ATP8A1 whereas other cells such as hippocampal neurons require ATP8A1 to maintain PS asymmetry.

Studies of ATP11C-deficient mice have provided strong evidence for a crucial role of ATP11C in establishing PS asymmetry in developing erythrocytes (Yabas et al., 2014). ATP11C-deficient mice were found to display reduced uptake of fluorescently labeled PS by erythroblasts but not mature erythrocytes. However, circulating erythrocytes accumulated PS on their surface, displayed abnormal shape characteristic of stomatocytosis, and had a shortened lifespan. These studies indicate that ATP11C is critical in generating and maintaining PS asymmetry during erythropoiesis, a process that is important in generating normal mature erythrocytes. The high content of PS and PE on the cytoplasmic leaflet of erythrocytes may be important for the interaction of the spectrin-ankyrin cytoskeletal network with the plasma membrane required for maintaining the normal shape of erythrocytes whereas the absence of PS on the exoplasmic leaflet of erythrocytes is important in maintaining the normal lifespan of these cells. ATP11C mediated internalization of PS has also been shown to be crucial for differentiation of B lymphocytes (Siggs et al., 2011a; Yabas et al., 2011).

PS asymmetry is also important in blood coagulation (Lentz, 2003). In this case retention of PS on the cytoplasmic leaflet of platelets and other blood cells prevents blood coagulation from occurring (Lentz, 2003; Lhermusier et al., 2011). This asymmetry is established by one or more active P4-ATPases and an inactive scramblase. In a proteomic screen, several P4-ATPases have been identified in human platelets including ATP8A1, ATP8B2, ATP11A-C, and ATP9B (Lewandrowski et al., 2009). Whether all or only selective P4-ATPases contribute to PS asymmetry remains to be determined. Blood coagulation is initiated when the bidirectional scramblase TMEM16F is activated by an increase in the intracellular Ca^2+^ concentration leading to PS exposure on platelet cell surfaces (Suzuki et al., 2010; Lhermusier et al., 2011; Malvezzi et al., 2013; Brunner et al., 2014). As part of this process, P4-ATPases may also be inhibited directly or indirectly by an increase in intracellular Ca^2+^, although this remains to be determined. Coagulation factors including Factor V can bind to the surface exposed PS through their discoidin domains to initiate the blood coagulation cascade. Loss-of-function mutations in TMEM16F have been shown to cause Scott syndrome, a severe bleeding disorder supporting the role of this scramblase in PS exposure and the blood coagulation process (Lhermusier et al., 2011).

## PS-asymmetry in apoptosis

Apoptosis is another well-established process involving the interplay of P4-ATPases and scramblases (Segawa et al., 2014; Hankins et al., 2015). The exposure of PS on the surface of cells is an “eat me” signal for phagocytic cells to engulf the apoptotic cell. Xkr8, a member of the XK family of membrane proteins, has been implicated as the scramblase that exposes PS on apoptotic cells for engulfment by macrophages (Suzuki et al., 2013). ATP11C plays a key role in restricting PS to the inner leaflet of the plasma membrane of normal cells. Cleavage of ATP11C at multiple caspase sites can inactivate ATP11C thereby facilitating apoptosis. Mutations in ATP11C that prevent caspase cleavage, but retain PS transport activity, do not expose PS on the surface of cells and these cells are not ingested by macrophage (Segawa et al., 2014). However, ATP11C is not the only P4-ATPase which is important in preventing PS exposure on cell surfaces. In some cells ATP11A with a similar substrate specificity and plasma membrane localization as ATP11C can compensate for the loss of ATP11C and prevent exposure of PS on cell surfaces (Segawa et al., 2016). Like ATP11C, ATP11A has a number of caspase cleavage sites. These studies suggest that the activation of the scramblase together with inhibition of P4-ATPases may be required for exposure of PS on cells as a signal for apoptosis.

## P4-ATPases implicated in intracellular vesicle trafficking

In addition to playing a role in establishing phospholipid asymmetry in the plasma membrane, P4-ATPases also play a crucial role in generating phospholipid asymmetry in intracellular membranes, a process linked to vesicle trafficking in cells. Most membrane lipids are synthesized on the cytoplasmic side of the ER and redistributed symmetrically across the membrane by scramblases. Vesicles budding from the ER fuse with the Golgi complex. Lipid asymmetry is induced in the TGN through the action of active flippases and floppases. Most information on the role of P4-ATPases in vesicle trafficking comes from studies of the yeast P4-ATPase Drs2p by Graham and colleagues (Sebastian et al., 2012). Drs2p is localized to the TGN where it flips PS and PE to the cytoplasmic leaflet to generate aminophospholipid asymmetry (Hua et al., 2002; Natarajan et al., 2004). The flippase activity of Drs2p is thought to initiate membrane curvature by increasing PS and PE content on the cytoplasmic leaflet at the expense of the lumenal leaflet leading to membrane curvature as initially proposed in the coupled bilayer hypothesis of Sheetz and Singer (1974) and Xu et al. (2013). Membrane curvature together with the increase in the negatively-charged PS recruits Arf-GTPase-activating protein Gcs1 and clathrin coat proteins to the cytoplasmic leaflet as part of the vesicular transport process between the TGN and early endosomes (Sebastian et al., 2012).

P4-ATPases have also been implicated in vesicle budding and trafficking in other eukaryotic cells although the mechanism is poorly understood. ATP8A2 is crucial for axon elongation in PC12 cells and hippocampal neurons suggesting a role of this P4-ATPase in vesicle trafficking required for neurite extension (Xu et al., 2012). Similarly, ATP8A2 deficient mice show a striking loss in the length of photoreceptor outer segments suggesting that a decrease in vesicle trafficking between the inner and outer segment of these cells occurs in the absence of ATP8A2 (Coleman et al., 2014). More recently, Lee et al. have shown that ATP8A1-catalyzed flipping of PS in recycling endosomes is important in the recruitment of EHD1 to the cytoplasmic leaflet of recycling endosomes and membrane fission (Lee et al., 2015). Finally, ATP8A1 has been associated with cell migration (Kato et al., 2013).

## Role of P4-ATPases in human disease

To date, two P4-ATPases, ATP8B1 and ATP8A2, have been directly associated with human genetic disorders. Mutations in the *ATP8B1* are responsible for two related liver diseases, known as benign recurrent intrahepatic cholestasis type 1 (BRIC1) and the more severe form progressive familial intrahepatic cholestasis type 1 (PFIC1) (Bull et al., 1998). These early onset diseases are characterized by impaired bile flow or cholestasis. Affected individuals can also experience hearing loss and are more susceptible to pneumonia (Stapelbroek et al., 2009; Ray et al., 2010). ATP8B1 has been localized to the canalicular membrane where it is thought to function as a phospholipid flippase to maintain the integrity of the membrane (Folmer et al., 2009b). This is supported in studies defining the effect of ATP8B1 knockdown on cultured rat hepatocytes (Cai et al., 2009). Loss in ATP8B1 resulted in normal ABCB4 expression and localization to the canalicular membrane, but impaired bile secretion and aberrant microvilli morphology. In Caco-2 cells, loss in ATP8B1 expression also causes significant reduction in microvilli supporting a role of ATP8B1 in maintaining the apical membrane structure. The effect of missense mutations associated with PFIC1 and BRIC1 on the stability of ATP8B1 and its interaction with CDC50A was studied in transfected CHO cells (Folmer et al., 2009b). The mutants were found to be less stable than the wild-type proteins and show a loss or decreased interaction with CDC50A. Furthermore, the mutants were not detected on the canalicular membrane of WIF-B cells. The impaired targeting to the plasma membrane of ATP8B1 caused by the most frequent ATP8B1 disease mutation in European patients, I661T, was recently shown to be rescued by compounds known to be efficient correctors of CFTR misfolding in cystic fibrosis, thus pointing to a possible therapeutic strategy (Van Der Woerd et al., 2016). The mechanism by which loss in ATP8B1 leads to cholestasis is poorly understood, but it has been proposed that increased PS on the luminal leaflet on canalicular membranes due to the loss in ATP8B1 flippase activity results in abnormal lipid packing (Folmer et al., 2009a). This in turn makes the outer leaflet of the canalicular membrane susceptible to bile salt mediated extraction of cholesterol and phospholipids resulting in reduced activity of ABCB11, a major bile salt exporter that has also been linked to PFIC2 (Strautnieks et al., 1998). The recent studies suggesting that ATP8B1 is a PC and not a PS flippase, however, leaves this model open to further investigations. It is possible that deficiency in PC transport to the inner membrane of canalicular membranes due to mutations in ATP8B1 could compromise the stability of the membrane to bile salts. More studies are needed to further define the role of ATP8B1 in cholestasis.

Genetic defects in ATP8A2 have been reported in two families. One individual with mental retardation and hypotonia had a balanced translocation of chromosomes 10 and 13 which disrupted the coding sequence of the *ATP8A2* gene (Cacciagli et al., 2010). More recently, a missense mutation (I376M) was identified as the cause of a rare neurodegenerative disease known as cerebellar ataxia, mental retardation, and dysequilibrium syndrome (CAMRQ) in a consanguineous family from Turkey (Onat et al., 2012). The I376M mutation is present in a conserved region of the M4 transmembrane segment of ATP8A2. Biochemical characterization confirmed that this missense mutation results in the loss in ATP8A2 PS flippase activity, as further discussed below (Vestergaard et al., 2014). Two ATP8A2-deficient mouse models, wabbler-lethal (*wl*) mouse and ATP8A2 knockout mouse, show similar neurological abnormalities including body tremors and ataxia resulting from distal axonal degeneration of the spinal cord (Zhu et al., 2012; Coleman et al., 2014). The *wl* mouse has a deletion in exon 22 of *Atp8a2* resulting in the expression of an inactive protein (Zhu et al., 2012). In addition to these characteristics, ATP8A2 deficient mice exhibit a loss in visual and auditory function and degeneration of photoreceptor and spiral ganglion cells (Coleman et al., 2014). These studies indicate that ATP8A2 plays an essential role in the function and survival of many neuronal cells, possibly by contributing to vesicle trafficking in axons and related neurological structures.

More limited information is available on the role of other P4-ATPases in human diseases (Van Der Mark et al., 2013). ATP10A has been implicated in the control of insulin-mediated glucose uptake in mouse adipose tissue and skeletal muscle and is considered as a risk factor for Type 2 diabetes in African-Americans (Dhar et al., 2004, 2006). ATP11A has been suggested to be a predictive marker for prognosis of colorectal cancer (Miyoshi et al., 2010). ATP11C-deficient mice have been shown to exhibit multiple abnormalities including loss in B cell development, hyperbilirubinemia, hepatocellular carcinoma, and anemia (Siggs et al., 2011a,b; Yabas et al., 2011). It remains to be determined if loss of function mutations in human ATP11C cause similar disorders. ATP8B4 has been implicated in Alzheimer's disease based on its chromosomal localization and analysis of single-nucleotide polymorphisms (Li et al., 2008).

## Structural and mechanistic features of P4-ATPases

### Reaction cycle of P4-ATPases

The predicted domain structure and topology together with conserved motifs for phosphorylation and dephosphorylation (Figures 1, 2) argue in favor of a flippase mechanism related to the well-known mechanism of the P2-ATPase ion pumps Ca^2+^-ATPase and Na^+^/K^+^-ATPase. Indeed, like the P2-ATPases and, presumably, all other P-type ATPases, the P4-ATPases are phosphorylated in a vanadate-sensitive manner by ATP at the conserved P-domain aspartate (Ding et al., 2000; Lenoir et al., 2009; Bryde et al., 2010; Coleman et al., 2012). The reaction cycle has been examined in some detail for ATP8A2 (Coleman et al., 2012), showing that the phosphoenzyme exists in E1P and E2P states, and that the dephosphorylation of E2P is activated by the specific phospholipid substrates PS and PE, but not by PC (Figure 3A). The latter finding is reminiscent of the effect of K^+^ binding from the external side to the Na^+^/K^+^-ATPase (Post et al., 1972). The K^+^ activation of dephosphorylation is one of the key features of the coupling mechanism of the Na^+^/K^+^-ATPase that allows K^+^ translocation from the external side to the internal side of the plasma membrane (Figure 3C). The specific phospholipid substrate apparently acts on the P4-ATPase in a similar way, promoting dephosphorylation in association with the translocation of the lipid from the exoplasmic to the cytoplasmic leaflet of the lipid bilayer (Figure 3B). Approximately 50-fold higher ATPase activity is observed for ATP8A2 in lipid vesicles containing 10% PS/90% PC as compared with 100% PC, where the ATPase activity is diminutive, and with PE the activation is also substantial (Coleman et al., 2009, 2012; Coleman and Molday, 2011). Crystal structures of the P2-ATPase ion pumps Na^+^/K^+^-ATPase and Ca^2+^-ATPase have shown how the conserved glutamate of the A-domain TGES motif (in P4-ATPases DGET) is brought into the correct position for catalyzing the hydrolysis of the aspartyl phosphoryl bond in the P-domain. This unique event consists of a 90° rotation of the A-domain in relation to the E1P–E2P transition followed by an additional, smaller change of conformation elicited by the binding of the ion to be transported from the exoplasm toward the cytoplasm (Toyoshima, 2009; Palmgren and Nissen, 2011). Similar structural changes are expected to result in dephosphorylation of P4-ATPases. Accordingly, it was demonstrated for ATP8A2 that mutation of the conserved A-domain glutamate blocks dephosphorylation (Figure 3A; Coleman et al., 2012). It is noteworthy that the structural information from the P2-ATPases shows that the catalytic site at which dephosphorylation occurs is located some 50 Å from the membrane site at which K^+^ binds to promote the dephosphorylation. In P4-ATPases the corresponding distance is likely no less, with the catalytic site being composed of the cytoplasmic A-, P-, and N-domains, and the lipid substrate coming from the exoplasmic leaflet, thus indicating that the signal transduction must take place through a conformational change reaching over long distance.

### Kinetic difference between ATP8A2 and Drs2p

Dephosphorylation of the yeast homolog of ATP8A2, Drs2p, is also accelerated by PS, but to a lesser extent than the dephosphorylation of ATP8A2 and only in the simultaneous presence of phosphatidylinositol-4-phosphate binding to the C-terminal regulatory domain (Jacquot et al., 2012). This observation is in accordance with the evidence mentioned above that Drs2p requires such interaction for PS flippase activity, thus being autoinhibited by the C-terminus in the absence of the activator. The C-terminus likely interferes with the optimal positioning of the A-domain for catalysis of dephosphorylation (Montigny et al., 2016). However, even in the presence of phosphatidylinositol-4-phosphate the PS-activated ATP hydrolysis and dephosphorylation rates of Drs2p are 1–2 orders of magnitude lower than those of ATP8A2 (Jacquot et al., 2012). The ATP hydrolysis and dephosphorylation rates determined for ATP8A2 are comparable to the rates seen for Ca^2+^-ATPase and Na^+^,K^+^-ATPase (Coleman et al., 2012). It has been suggested that the high catalytic turnover rate of ATP8A2 is needed particularly in the retinal membranes housing ATP8A2, due to rapid dissipation of lipid asymmetry by the floppase activity of the retinal protein opsin (Jacquot et al., 2012). However, ATP8A2 is expressed not only in the retina, but all over the brain, where it is indispensable, as evidenced by the CAMRQ syndrome resulting from a single missense mutation of ATP8A2. Even in the presence of its known activator Drs2p appears more autoinhibited than ATP8A2. Worth noting in this connection is the fact that there is only poor conservation between ATP8A2 and Drs2p of the C-terminal tail including the putative RMKKQR phosphatidylinositol-4-phosphate-binding site (Jacquot et al., 2012). More information that possibly can be obtained by studying the consequences of exchanging their C-termini is needed to resolve this issue. The coupling between ATP hydrolysis and lipid flipping should also be considered. The stoichiometry of PS molecules flipped per ATP molecule hydrolyzed has not been determined with confidence, one reason being that the ATPase activity is rather low with the NBD-labeled PS used for measurement of transport. It is thus an open question whether ATP8A2 hydrolyzes more ATP per lipid flipped than Drs2p.

### Phosphorylation of P4-ATPases does not need triggering by ions being countertransported

In the Na^+^/K^+^-ATPase and the Ca^2+^-ATPase, the binding of the ion being transported from the cytoplasmic to the exoplasmic side triggers the phosphorylation from ATP (Figure 3C). It is therefore relevant to ask whether the analogy between P4-ATPase and P2-ATPase catalytic mechanisms further implies that the binding of another specific substrate transported in the reverse direction of the known lipid substrates, from the cytoplasmic to the exoplasmic side, is needed to activate the phosphorylation of the P4-ATPase from ATP. This question has been addressed by examining the effects of various ions including Na^+^, K^+^, Cl^−^, ${\text{SO}}_{4}^{2-}$, Ca^2+^, N-methyl-D-glucamine, acetate, and H^+^ on the phosphorylation of ATP8A2 from ATP, with the result that none of these ions promote phosphorylation at a significantly higher rate than the others (Coleman et al., 2012). Similar results were obtained for Drs2p in a study that tested in addition to the ions mentioned above, Co^2+^, Zn^2+^, La^3+^, carbonyl cyanide, m-chlorophenylhydrazone, oxalate, and arsenate, in all cases without finding appreciable effects on phosphorylation (Jacquot et al., 2012). Perhaps the most likely candidate for a non-lipid substrate transported by the P4-ATPases toward the exoplasm would be H^+^, but rapid kinetic analysis of the pH dependence of the phosphorylation rate showed no significant variation in the interval from pH 6.5 to pH 9.0, thus indicating that if proton binding at a specific site (supposedly followed by transport) really were required for activation of the phosphorylation reaction, then the proton affinity of this site would be unusually high with deprotonation occurring only above pH 9.0 (Coleman et al., 2012). The conclusion of these studies is therefore that presently there are no ions known as activators of the phosphorylation of P4-ATPases except for Mg^2+^, which is required as catalyst at the catalytic site as for other P-type ATPases. The apparent affinity for Mg^2+^ activation of phosphorylation is in the micromolar range and very similar to that pertaining to Na^+^/K^+^-ATPase (Coleman et al., 2012).

### Pathway for phospholipid movement studied by mutagenesis and structural modeling

#### Giant substrate problem

While the catalytic mechanism of ATP hydrolysis by P4-ATPases appears similar to that of the P2-type ATPases, these ion pumps might be less suitable models for the P4-ATPases when it comes to understanding the handling of the lipid substrate. The size of the ions being transported by the P2-ATPases is only 2–3 Å in diameter, allowing occlusion of the ions in a narrow space between the transmembrane helices M4, M5, M6, and M8 during their passage across the membrane. Phospholipids are much larger (the hydrocarbon chains are approximately 20 Å long and the head group 5 Å), taking up space corresponding to half the membrane bilayer thickness, and must reorient during the flipping to keep the hydrophilic head group at the bilayer surface. The large size of the lipid substrate and the complex movement it has to undergo make it hard to imagine a common transport mechanism for ions and lipids. Solving this enigma, the so-called “giant substrate problem,” is a most essential challenge of P4-ATPase research. Because flippase activity is retained following purification and reconstitution of ATP8A2 together with its accessory CDC50A subunit in lipid vesicles (Coleman et al., 2009, 2012; Coleman and Molday, 2011), there is no doubt that these are the only protein components required for the movement of the lipid and its coupling with ATP hydrolysis, thus providing a sound basis for using site-directed mutagenesis to search for residues interacting with the lipid substrate during the flipping. Mutational effects on the apparent affinity for the lipid substrate can be assessed from the PS or PE concentration dependences of the ATPase activity and/or the dephosphorylation step. PS or PE is added at various concentrations to the ATP8A2-CDC50A complex maintained solubilized in detergent and PC. This procedure first pinpointed the lysine K873 as critical for the interaction with the lipid substrate (Coleman et al., 2009; Coleman and Molday, 2011; Coleman et al., 2012). The K873A and K873E mutants showed conspicuous reductions of the apparent affinity for PS in ATPase activity studies as well as in studies of the PS-activated dephosphorylation, whereas a more moderate reduction was seen for K873R, thus indicating a role of the positive charge of the side chain, which might be to interact with a negative charge of the phospholipid head group. Because the binding of PE was disturbed as well by the mutation, the suggested interaction would implicate the phosphate part of the head group common to both lipids (Coleman et al., 2012). K873 is located in the M5 transmembrane helix at exactly the position corresponding to a serine in Na^+^/K^+^-ATPase that is a key residue in the coordination of K^+^ during the transport (Blostein et al., 1997; Pedersen et al., 1998; Shinoda et al., 2009). Moreover, significant effects were observed for mutation of the adjacent N874, which corresponds to an asparagine that participates in the coordination of K^+^ in Na^+^/K^+^-ATPase and Ca^2+^ in the Ca^2+^-ATPase (Coleman et al., 2012), see the sequence alignment in Figure 2. Thus, the similarity between the P4-ATPase and the P2-ATPases clearly reaches beyond the mechanism of ATP hydrolysis, managed by the cytoplasmic domains, to the events taking place in the membranous part of the protein.

Would it really be feasible that the giant phospholipid substrate is translocated through a pathway in the protein that involves occlusion between the transmembrane helices M4, M5, M6, and M8 similar to the occlusion of ions being translocated by the Na^+^/K^+^-ATPase and the Ca^2+^-ATPase? After all, this possibility might not be totally unrealistic, because the crystal structure of another related P-type ATPase ion pump, the plant H^+^-ATPase AHA2 (P3-ATPase), unlike the crystal structures of the Na^+^/K^+^-ATPase and the Ca^2+^-ATPase, shows the presence of a large central cavity containing water (Pedersen et al., 2007). The existence of this cavity is possibly the consequence of the absence in the H^+^-ATPase transmembrane helices of most of the negatively charged residues that in Na^+^/K^+^-ATPase and Ca^2+^-ATPase cross-link the helices through the binding of the transported cations. These carboxylic acid residues are also absent in the P4-ATPases, being replaced by hydrophobic residues in several cases (Figure 2). Hence, as long as structural details of P4-ATPases in the membrane region remain unknown, the option of a central cavity that might accommodate a lipid temporarily during the flipping needs to be taken into consideration. This hypothesis has also been referred to as a “canonical binding site” (Baldridge and Graham, 2013), because the lipid being flipped would be located similarly to the occluded ions in the “classic” ion pumps.

#### Possible peripheral pathway

The alternative, and at the moment perhaps most realistic, transport model is one in which a peripheral pathway in the protein allows for flipping of the phospholipid in such a way that only the polar head group interacts directly with the protein. This would allow the hydrocarbon tail to slide through the hydrocarbon layer of the lipid bilayer surrounding the protein, sometimes referred to as the “credit card model” because the interaction of the protein exclusively with the head group is comparable to a credit card payment terminal, which touches only the magnet stripe or chip of the card. The relevance of such a peripheral pathway type of model is underscored by the recent publication of the crystal structure of a scramblase, TMEM16 (Brunner et al., 2014). The scramblase equilibrates PS and PE phospholipids between the two bilayer leaflets and contains at its lipid bilayer exposed surface an 8–11 Å wide crevice spanning the entire membrane. This crevice is formed by transmembrane helices and is twisted like a spiral staircase with one side exposed to the membrane. Its surface is strongly hydrophilic, thus being able to accommodate the head group of the phospholipid being flipped, with the hydrocarbon tail extending into the surrounding lipid bilayer in accordance with the “credit card model.”

#### Non-canonical peripheral pathway model

A clue to where in the P4-ATPase structure a peripheral pathway might be located was first obtained in studies of the yeast P4-ATPases Dnf1p and Drs2p (Baldridge and Graham, 2012, 2013). The different lipid substrate specificities of these flippases (PC and PS, respectively) allowed identification of amino acid residues with a role in defining the substrate specificity by examining chimeras and point mutants in which residues had been exchanged between Dnf1p and Drs2p. Critical residues were pinpointed in the membrane region, particularly a M3 asparagine (N550 of Dnf1p corresponding to N302 in bovine ATP8A2) and a M4 phenylalanine/tyrosine (Y618 of Dnf1p corresponding to L367 in bovine ATP8A2), as well as in M1 near its exoplasmic end. Supposedly residues contributing to define the selectivity for PC versus PS would be near a pathway for translocation of the phospholipid head group, and the locations of the pinpointed residues are not consistent with a central, canonical binding site. Consideration of a structural model of Dnf1p based on the crystal structure of the Ca^2+^-ATPase in the E1 state led to the conclusion that these residues face the surrounding lipid phase in accordance with a peripheral translocation pathway involving mainly M1, M3, and M4, which was denoted as the “non-canonical pathway” (Baldridge and Graham, 2012, 2013).

#### Hydrophobic gate peripheral pathway model

More recently, a slightly different location of a putative peripheral pathway was suggested by structural modeling prompted by mutagenesis results for other residues in M4, particularly the isoleucine in the middle of M4 (Vestergaard et al., 2014). This isoleucine is part of the PISL motif (Figure 2), which is conserved among P4-ATPases. Significantly, the isoleucine is located exactly at the position corresponding to the glutamate in the corresponding PEGL motif of the Ca^2+^-ATPase and the Na^+^/K^+^-ATPase. Like P4-ATPases, P3-ATPases such as the AHA2 mentioned above have either isoleucine or valine at the corresponding position (Figure 2). On the basis of mutagenesis work in combination with structural information from crystallization, the glutamate present in the Na^+^/K^+^-ATPase and the Ca^2+^-ATPase is known to be a key element in the transport mechanism. It works as a gating residue at the ion binding pocket and furthermore directly binds and moves the ions undergoing transport, being alternately exposed toward the two sides of the membrane during the pump cycle. Hence, in these P2-ATPase ion pumps M4 moves vertically like a pump rod, allowing delivery to the exoplasm of Na^+^ or Ca^2+^ bound at the M4 glutamate from the cytoplasmic side, or delivery to the cytoplasm of K^+^/H^+^ bound at the same glutamate from the exoplasmic side (Vilsen and Andersen, 1998; Olesen et al., 2007; Toyoshima, 2009). It was therefore intriguing to learn that a missense mutation replacing this isoleucine (I376 in human ATP8A2) with methionine had been identified as the first (and so far only) disease-causing mutation in ATP8A2, giving rise to CAMRQ (Onat et al., 2012). When expressed in mammalian cell culture the equivalent mutation in bovine ATP8A2, I364M, disrupted flipping of fluorescent NBD-labeled PS and reduced ATPase activity to very low levels, although wild type-like expression levels were retained. Analyses of the lipid substrate concentration dependence of the overall and partial reactions of the enzyme cycle in ATP8A2 mutants with various replacements of this isoleucine led to the conclusion that during the lipid flipping the PS head group passes near I364, and I364 is critical to the release of the lipid into the cytosolic leaflet (Vestergaard et al., 2014).

**Figure 4:**
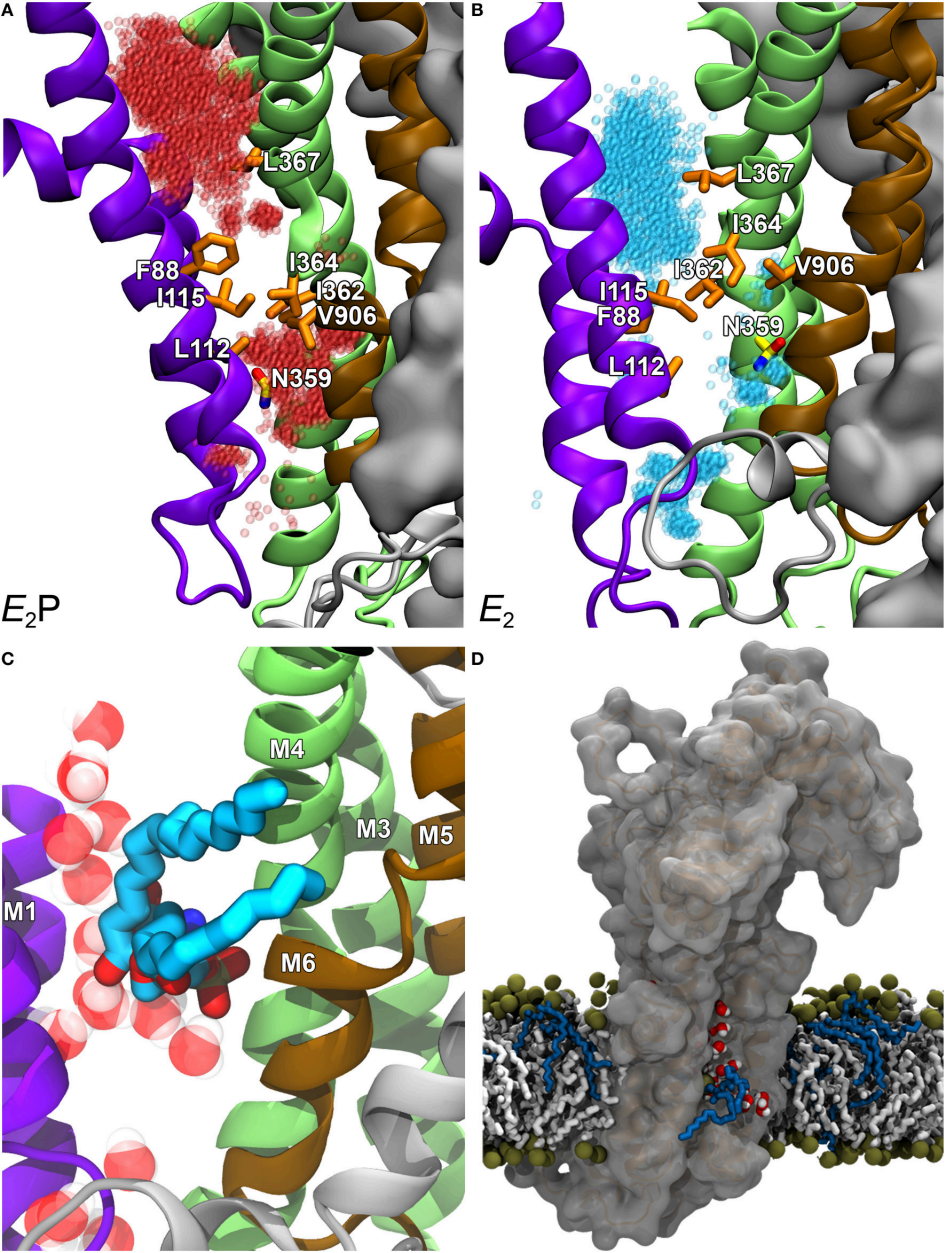
**Structural model of ATP8A2 in E_2_P and E_2_ states revealing the hydrophobic gate pathway**. The model was constructed on the basis of the alignment of ATP8A2 with SERCA1a shown in Figure 2 and the crystal structures of SERCA1a in E_2_P and E_2_ states supplemented with refinement of the membrane domain by molecular dynamics simulation, opening up a groove between M1, M2, M4, and M6 (Vestergaard et al., 2014). In all panels, the cytoplasmic side is up. **(A,B)** Refined models of E_2_P and E_2_ states are shown in cartoon with helices M1–M2 in purple, M3–M4 in green, M5–M6 in brown, and M7–M10 as molecular isosurface representation in gray. Selected hydrophobic side chains including I364 are shown in licorice representation with carbons in orange for hydrophobic residues and in yellow for N359, nitrogen in blue, and oxygen in red. Water molecules in groove are red transparent spheres in the E_2_P model and cyan in the E_2_ model. **(C)** Metadynamics simulations were used to steer a phosphatidylserine molecule into the groove of the E_2_ model. **(D)** Visualization of credit card transport hypothesis. E_2_ model in surface representation embedded in the phospholipid membrane, where brown spheres indicate phosphate parts of the phospholipid head groups and hydrocarbon chains are shown in white (PC) or blue (PS) licorice representation. The hydrophilic head group of a PS molecule is being moved upwards along the groove, partly solvated by water molecules (oxygen red, hydrogen white). The blue hydrocarbon chains of the lipid project into the membrane lipid phase.

To examine the exact role of I364, in the absence of a crystal structure of a P4-ATPase, homology-based models of ATP8A2 were built for E2P and E2 (the two states that interact with the lipid substrate, cf. Figure 3B), starting from a multiple sequence alignment and the high-resolution crystal structures of the Ca^2+^-ATPase in these conformations. The models were subsequently refined by molecular dynamics simulation, which relaxed the original ATP8A2 homology model structures to structures where a groove between the M1, M2, M4, and M6 transmembrane helices had opened to a wider state, thus suggesting that this region, now deviating significantly from that of the P2-ATPase ion pumps, might be directly involved in lipid transport by the P4-ATPases (Vestergaard et al., 2014). Substantial amounts of water entered the groove during the molecular dynamics refinement, gathering in two distinct pockets placed in the exoplasmic and cytoplasmic ends of the groove, separated by a cluster of hydrophobic residues that included I364 (Figures 4A,B). This hydrated groove has a size appropriate for accommodation of the PS head group and is located rather peripherally in the protein, thus allowing the hydrocarbon tail of the lipid to project out into the surrounding membrane bilayer lipid during the flipping (Figures 4C,D), exactly as proposed for the scramblase TMEM16 described above. In the P4-ATPase, the phospholipid head group in the groove would be solvated by the water in the groove, thereby aiding the movement of the head group. An important feature of the ATP8A2 E2P and E2 structural models is moreover that I364 moves during the transition between these states, thereby apparently functioning as a “hydrophobic gate” propelling the water in the groove with the solvated phospholipid head group toward the cytoplasmic side against the concentration gradient.

**Figure 5:**
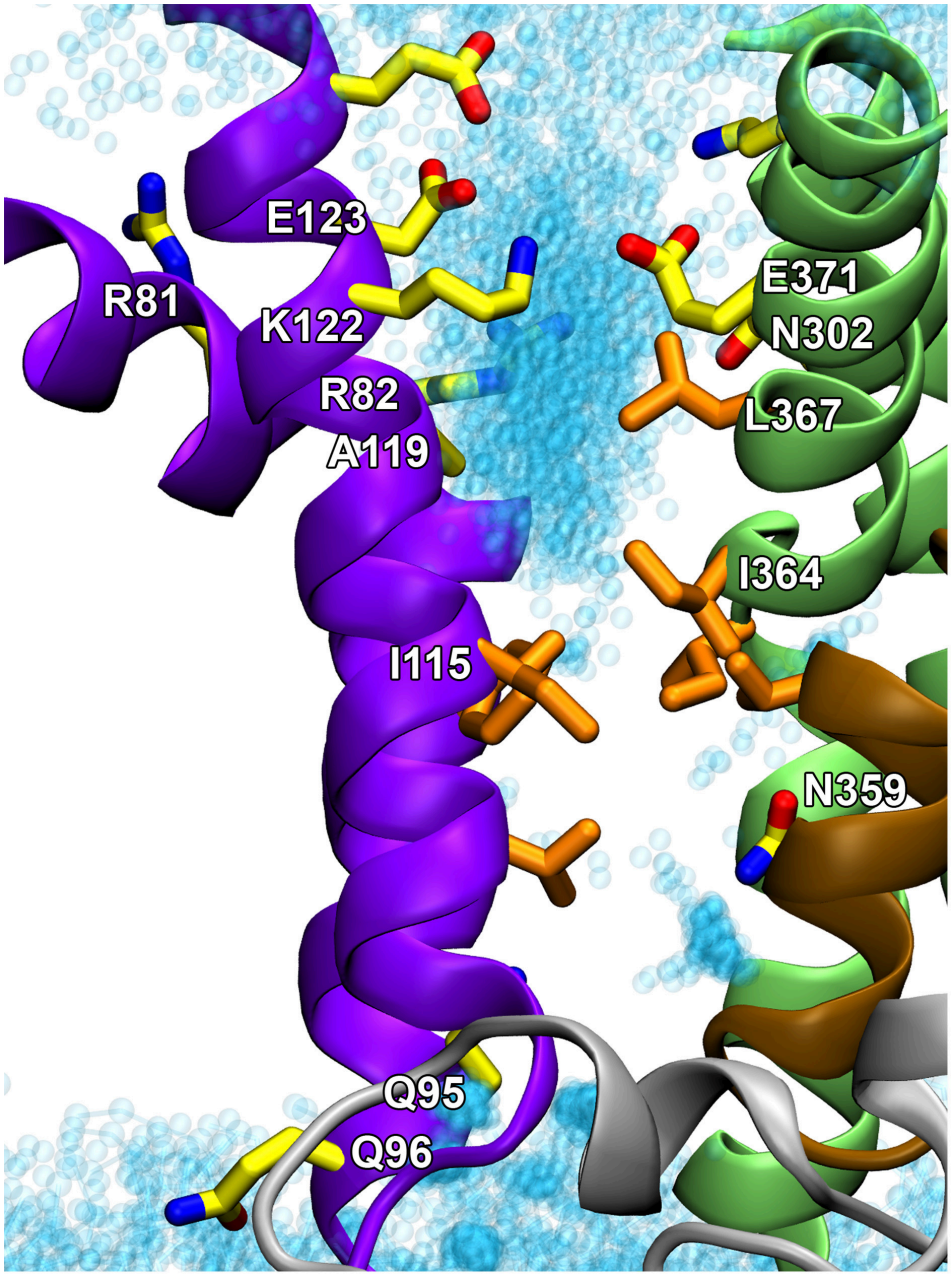
**Proposed hydrophobic gate pathway in side view with indication of residues that might play a role in the recognition and translocation of the phospholipid**. The E2 structural model of the ATP8A2 membrane domain (Vestergaard et al., 2014) is visualized with the cytoplasmic side up. Selected residues, some of which are mentioned in the text, are shown in licorice representation with carbons in orange for hydrophobic residues and in yellow for polar/charged residues. Water molecules in the groove are shown as cyan transparent spheres. I364 and I115 are key residues in the hydrophobic gate. N359 is important for the affinity for the lipid substrate (Vestergaard et al., 2014). Q95, Q96, A119, E123, N302, and L367 are located at positions corresponding to the yeast Dnf1 flippase residues G230, A231, T254, D258, N550, and Y618, which have been shown to be crucial to the specific lipid selectivity of this flippase (Baldridge and Graham, 2012, 2013).

Mutational analysis further implicated adjacent hydrophobic residues including I115 (of M2) as part of the gate (Vestergaard et al., 2014). The polar residue N359 (of M4) appears to be involved in the recognition of the lipid substrate entering the groove from the exoplasmic side (**Figures 4A,B, 5**). These findings suggest a plausible phospholipid translocation mechanism that is both distinct from and related to the well-characterized P2-ATPase ion transport mechanisms. In lipid pumps as well as ion pumps, M4 plays a central role as the pump rod generating the movement. In fact, the less wide groove present between M1, M2, M4, and M6 in the Ca^2+^-ATPase also seems to contain water and has been proposed to be the pathway taken by Ca^2+^ migrating from the cytoplasmic side to the Ca^2+^ occlusion pocket (Musgaard et al., 2012), thus overlapping partially with the hydrophobic gate pathway proposed for ATP8A2 (Vestergaard et al., 2014). In a recent review (Montigny et al., 2016) it was furthermore pointed out that the most conserved of the two Ca^2+^ binding sites found in Ca^2+^-ATPases is the one involving the glutamate in M4 corresponding to I364 of ATP8A2, denoted site II in SERCA, implying that Ca^2+^-ATPases like PMCA and SPCA that only transport one Ca^2+^ in each cycle do not possess a site corresponding to site I of SERCA, and therefore do not use residues in M5 and M8 for Ca^2+^ binding. Hence, the hydrophobic gate pathway must be very similar to the transport pathway used by the one-site Ca^2+^-ATPases and possibly also other P-type ATPases pumping a single ion per cycle (Montigny et al., 2016). As previously alluded to in discussing the relation between P2- and P4-ATPases (Lenoir et al., 2007; Baldridge et al., 2013), the Ca^2+^-ATPase E2 crystal structure shows the presence of a phospholipid molecule approximately corresponding to the cytoplasmic exit of the hydrophobic gate pathway (top of Figure 5). In the P4-ATPases, this primordial phospholipid-binding site might have evolved into a site playing a role in the lipid transport. Worth mentioning in relation to the conservation of the structure and basic mechanism between P2- and P4-ATPases is also the proline adjacent to the isoleucine in the PISL motif (Figure 2). This proline is conserved in all P-type ATPases, and in ATP8A2 the P363A mutation abrogated expression of the protein in HEK cells, suggesting that the local unwinding of the M4 helix near PISL caused by the presence of the proline is crucial to proper folding and processing of the protein (Vestergaard et al., 2014).

#### Merge of structural models resulting from ATP8A2 and yeast flippase studies of the lipid transport pathway

**Figure 6:**
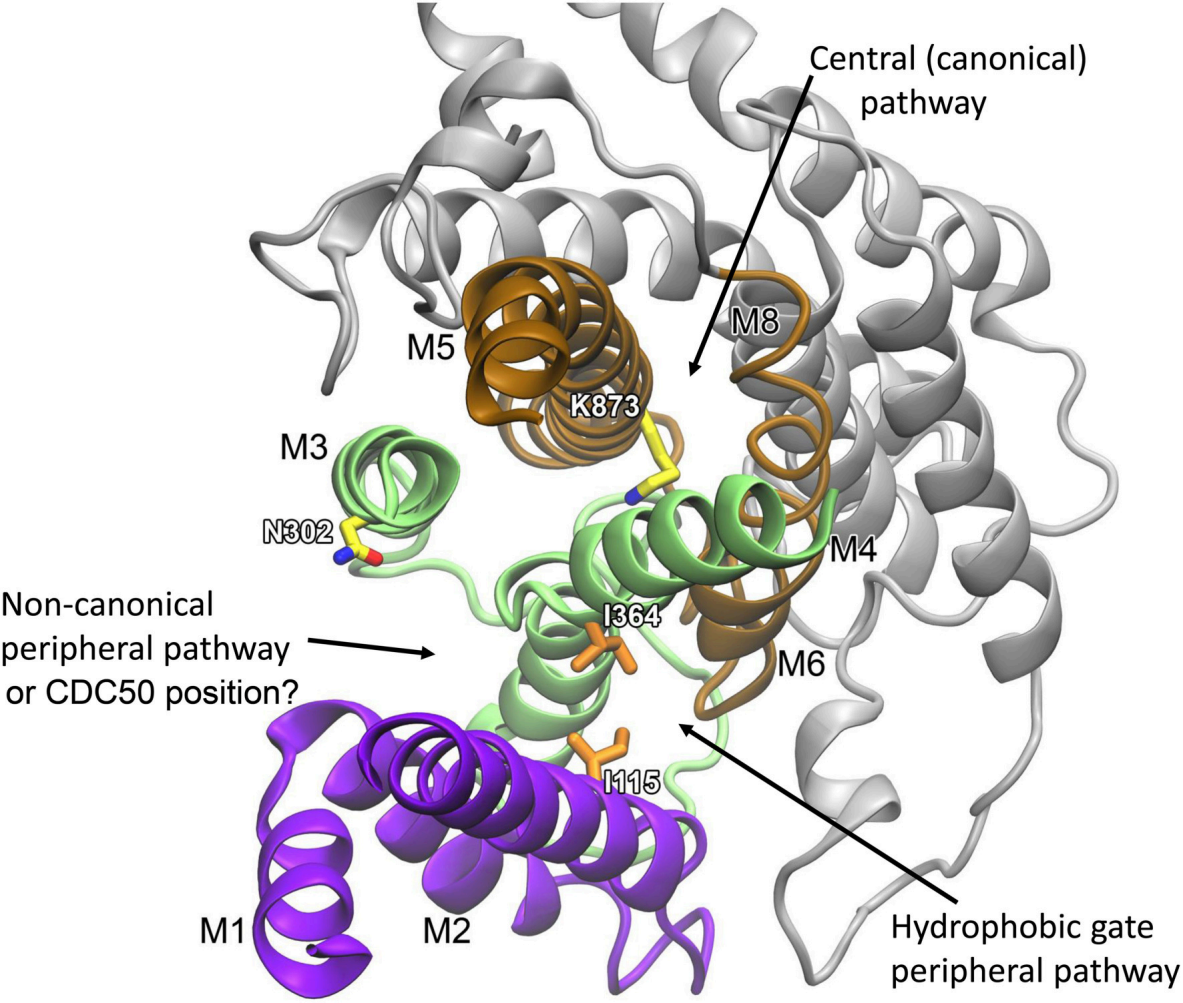
**Overview of the locations of three proposed phospholipid transport pathways and possible location of CDC50**. The refined membrane domain of the E2 structural model of ATP8A2 (Vestergaard et al., 2014) is visualized as viewed from the cytoplasmic side. Helices M1–M2 are shown in purple, M3–M4 in green, M5–M6 in brown, and M7–M10 in gray. Selected residues mentioned in the text are shown in licorice representation with carbons in orange for hydrophobic residues and in yellow for polar/charged residues. Recent evidence suggests a location of the CDC50 protein corresponding to the non-canonical peripheral pathway (Vestergaard et al., 2015).

The structural locations of the various proposed P4-ATPase lipid translocation pathways are summarized in Figure 6: the peripheral pathway associated with the hydrophobic gate (between M1, M2, M4, and M6; Vestergaard et al., 2014); the central pathway implying occlusion of the lipid between M4, M5, M6, and M8 similar to the occlusion of ions in the two-sites Ca^2+^-ATPases and the Na^+^,K^+^-ATPase; and the non-canonical pathway between M1, M3, and M4 (Baldridge and Graham, 2012, 2013). It is of note that in the structural model of Vestergaard et al. (2014), the M4 residue L367, which is equivalent to Y618 shown to be important for lipid head group selectivity in yeast Dnf1p (Baldridge and Graham, 2012, 2013), points toward the hydrophobic gate pathway, being located one helix turn above I364 (Figure 5). Likewise, the M2 residues A119 and E123 at positions equivalent to yeast Dnf1 residues T254 and D258 shown to couple with the M4 Y618 to confer lipid head group selectivity (Baldridge et al., 2013), seem in the structural model of Vestergaard et al. (2014) to be associated with the cytoplasmic exit of the hydrophobic gate pathway (Figure 5). On the other hand, N302, equivalent to the asparagine N550 also pinpointed as critical for head group selectivity in the yeast flippase studies (Baldridge and Graham, 2013), is too distant from the hydrophobic gate pathway to be directly involved (Figures 5, 6).

It is clear that in addition to the hydrophobic gate, residues on its cytoplasmic side pointing toward the proposed translocation pathway contribute to determining the affinity for the lipid substrate. The yeast flippase studies have furthermore identified two residues near the exoplasmic end of M1 that are important for lipid substrate recognition (Baldridge and Graham, 2013). At the equivalent positions of ATP8A2 are Q95 and Q96 shown in Figure 5. These residues might be associated with the exoplasmic entrance to the hydrophobic gate pathway. The separation of the residues critical to lipid substrate recognition in two clusters, one near the exoplasm and the other near the cytoplasm, observed in the yeast flippase studies, led to the view that there are two gates, an entry gate, and an exit gate (Baldridge and Graham, 2013). In fact the studies of ATP8A2 indicate that there are residues critical to the interaction with the lipid substrate all along the hydrophobic gate pathway. It is, however, not clear why residues near the cytoplasmic exit of the putative translocation pathway should be as critical for the lipid substrate recognition as the residues at the inlet. One possibility is that residues near the exit form bonds (note in Figure 5 that K122 and E371 are very close, probably forming a salt bridge) required to position the helices correctly for entry and recognition of the lipid substrate at the other end of the groove.

#### Role of the M5 lysine

In light of the proposal of the hydrophobic gate pathway one might wonder about the role of the more centrally placed critical lysine K873 of transmembrane helix M5 discussed above. In our refined models, K873 is situated on the opposite side of M4 with respect to the hydrophobic cluster including I364 and does not point into the suggested transport pathway (Figure 6). K873 is closely surrounded by polar residues with which it may form hydrogen bonds, including N905 located at the position corresponding to an ion binding asparagine/aspartate of Ca^2+^-ATPase and Na^+^/K^+^-ATPase (Figure 2), and this part of the models was most stable during refinement. K873 might therefore acts as a stabilizer of the central part of the structure, aiding the correct placement of the dynamic regions including M4 and the hydrophobic gate.

#### Structural location of the CDC50 protein

The “dark horse” in the modeling studies, so far not taken properly into consideration, is the CDC50 protein (the β-subunit). Recently, screening of 141 point mutations of the ATP8A2 catalytic subunit pinpointed six mutations that indirectly affect the glycosylation of the CDC50 protein, suggesting the possibility of a defect in the structural interaction between the two subunits of the complex (Vestergaard et al., 2015). Interestingly, in our structural model four of the six residues implicated are situated above each other almost along a line at the periphery of the ATP8A2 protein, pointing toward the putative location of the non-canonical pathway, between M1, M3, and M4 (Figure 6). Hence, one or both transmembrane helices of CDC50 might actually be located corresponding to the non-canonical pathway, which would place the CDC50 exoplasmic domain in close proximity to the putative exoplasmic entrance of the hydrophobic gate pathway, thereby enabling CDC50 to directly promote substrate binding in the hydrophobic gate pathway.

## Conclusions and future perspectives

Cell-based and biochemical studies from a number of research groups have convincingly shown that P4-ATPases actively flip phospholipids from the exoplasmic to the cytoplasmic leaflet of the plasma membrane and intracellular membranes. Like yeast P4-ATPases, some members of the mammalian P4-ATPase family actively transport the PS and PE to generate and maintain lipid asymmetry, whereas other members appear to be specific for PC. Cell culture and animal studies in which specific P4-ATPases are overexpressed or knocked down support the importance of these mammalian P4-ATPases in generating and maintaining lipid asymmetry for such key cellular processes as vesicle budding and trafficking, cell migration, neurite extension and cell survival. Although, significant progress has been made, a number of questions and areas of investigation remain. The distribution of P4-ATPases in native tissues and cells and their role in various biochemical pathways need to be studied in more detail. The substrate specificity of several mammalian P4-ATPases including ATP9A and ATP9B remain to be determined. Whether these proteins require an auxiliary protein to complete the flippase complex also remains to be determined. The mechanisms for regulation and subcellular targeting of mammalian P4-ATPases have yet to be examined in detail. We still know relatively little about the individual CDC50 proteins and their role in lipid flippase activity. Molecular modeling together with site-directed mutagenesis has provided important insight into how P4-ATPases may recognize and pump specific phospholipid substrates across membranes. Although, the lipid substrates of P4-ATPases are “giant” in comparison with the ions translocated by ion pumps, similarities between lipid and ion pumps of P-type greatly outnumber their differences. Not only are the mechanisms for ATP hydrolysis the same, but even the handling of the lipid substrate has much in common with the pumping of ions, and the suggested transport pathways of P-type lipid pumps and ion pumps appear to overlap to some extent. The hydrophobic gate pathway hypothesis should be validated by additional mutational studies, with the aim of identifying the determinants of the specific substrate selectivity of the P4-ATPases. For example, is there a “selectivity gate” at the exoplasmic entrance of the pathway, perhaps involving the CDC50 protein? A full understanding of the mechanism will require determination of the structure of a P4-ATPase with its bound lipid substrate. The highly purified ATP8A2 preparation recently developed seems optimal for such studies.

## Author contributions

JA, AV, SM, LM, MC, and RM have contributed to the writing and editing of this review.

## Funding

This study was funded in part by grants from the Danish Medical Research Council, the Novo Nordisk Foundation (Fabrikant Vilhelm Pedersen og Hustrus Legat), and the Lundbeck Foundation, and grants from the Canadian Institutes for Health Research (MOP-106667) and the National Institutes of Health (EY02422).

### Conflict of interest statement

The authors declare that the research was conducted in the absence of any commercial or financial relationships that could be construed as a potential conflict of interest.
